# Lunar cycling in sleep and mood in individuals with bipolar disorder

**DOI:** 10.1186/s40345-022-00282-4

**Published:** 2022-12-18

**Authors:** Helen J. Burgess, David Kagan, Samuel Warshaw, Zoey Jopling, Muneer Rizvydeen, Peisong Han, Anastasia K. Yocum, Melvin G. McInnis

**Affiliations:** 1 Department of Psychiatry, Sleep and Circadian Research Laboratory, University of Michigan, Rachel Upjohn Building, 4250 Plymouth Rd, Ann Arbor, MI 48109 USA; 2 Department of Biostatistics, School of Public Health, University of Michigan, Ann Arbor, MI 48109 USA; 3 Depression Center, Department of Psychiatry, University of Michigan, Ann Arbor, MI 48109 USA

**Keywords:** Actigraphy, Light, Moon

There is evidence that the lunar illuminance cycle impacts sleep and mood in humans. A cross-sectional study in healthy young participants using polysomnography found that on average there was a loss of 20 min of sleep near the full moon, as compared to near the new moon (Cajochen et al. 2013). In participants living in an urban setting, longitudinal wrist actigraphy recordings revealed that in the top quartile of “lunar cyclers”, sleep started on average 32 min later and sleep duration shortened by an average of 58 min near the time of the full moon versus the new moo (Casiraghi et al. 2021). In people living with bipolar disorder, previous reports have described changes in mood that associate with the lunar cycle (Wehr 2018). In many cases, there is a more positive mood near the full moon, and a more negative mood near the new moon, in accordance with the ~ 29.5 day lunar illuminance cycle. Recently we reported on a rapid cycling individual with bipolar disorder I whose daily ratings over 3.3 years indicated his sleep and mood aligned with the lunar cycle – with shorter sleep and more positive mood around the full moon, as compared to around the new moon (Burgess et al. 2021). This individual and his family were aware of the influence of the lunar cycle on his sleep and mood.

Recently, we enrolled 9 individuals with bipolar disorder I from the Heinz C. Prechter Longitudinal Study of Bipolar Disorder (McInnis et al. 2018) into a pilot study to further explore lunar cycling in bipolar disorder. These individuals had no other mental or physical illness, did not use prescription hypnotics or over-the-counter sleep aids including melatonin, did not use NSAIDs or beta-blockers on a daily basis (they suppress melatonin), reported no shift work or travel across time zones in the past month nor during the study, were not pregnant, and had no upcoming events that could impact their sleep. All participants but 1 passed a urine toxicology screen just prior to participation (participant P6 tested positive for THC), and no data collection occurred during the daylight savings transitions. Participant demographics are displayed in Fig. 1.

**Fig. 1:**
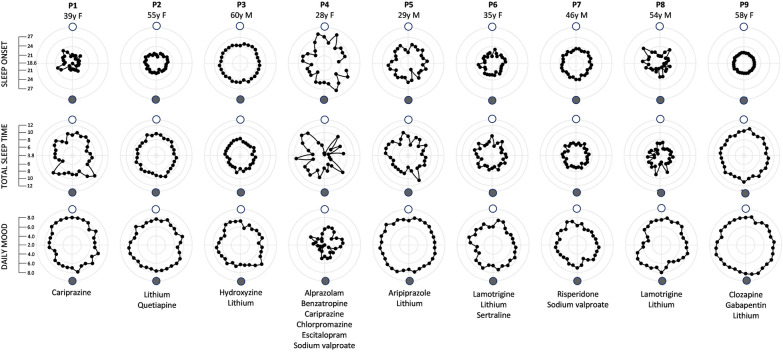
Circular plots of the average sleep onset time (clock time), total sleep time (hours), and daily mood ratings (1-worst mood to 10-best mood) for each participant, averaged across all available months relative to the lunar illuminance cycle. The full moon is shown as a white circle and the new moon as a gray circle. Each participant’s age and sex are listed at the top, with medications they were taking during the study listed below. Missing sleep data for P4 reflects this participant skipping sleep one night

In this pilot study, the participants’ nightly sleep was objectively measured with wrist actigraphy (Actiwatch Spectrum, data scored with Actiware 6.1.2. according to standard procedures (Patel et al. 2015)) and daily mood was tracked with time-stamped reports (1-worst mood to 10-best mood scale) for 35 days, ideally within each season. One individual participated for only 1 month (P1), three for 2 separate months (P4, P6, P8), two for 3 separate months (P2, P5), and three for 4 separate months (P3, P7, P9). Participants also collected overnight urine samples on the nights of the full and new moons (no alcohol or caffeine permitted 24 h prior), to explore possible changes in cortisol and the creatinine-adjusted melatonin metabolite during the lunar cycle (Cajochen et al. 2013; Dergaa et al. 2021), and changes in measures of DNA oxidative stress that are associated with melatonin levels (Davanipour et al. 2009). The study was advertised as tracking sleep across the seasons, and lunar cycling was not mentioned to any of the participants. This study was approved by the Institutional Review Board at the University of Michigan, and participants provided written informed consent.

The sleep onset time, total sleep time, and mood data for each participant in each month were plotted as circular plots using the statistical package R. Five-day averages, centered at the full and new moon respectively were calculated for each individual in each month. No effect of season was observed. When all available months for each participant were combined to generate a 5 day average centered at the full vs. new moon, no systematic influence of lunar phase on each of the three variables was observed in the group data (paired t-tests: sleep onset time, p = 0.27; total sleep time, p = 0.61; daily mood, p = 0.23). On an individual level, there were 2 participants who consistently showed a later sleep onset time around the full versus new moon across multiple months (P4, P5), starting sleep by 9 min to 2.8 h later near the full moon. However, only P4 showed a consistent reduction in total sleep time near the full moon, and neither participant showed a consistently more positive mood near the full versus new moon. Figure 1 displays sleep onset time, total sleep time, and daily mood for each participant, averaged across all available months relative to the full and new moon. The overnight urine collected in the first month was analyzed and did not reveal any systematic changes in cortisol, creatinine-adjusted melatonin metabolite, or DNA oxidative stress (8-oxo-7,8-dihydro-guanine, 8-oxo-7,8-dihydro-2′-deoxyguanosine) (paired t-tests p ≥ 0.29).

In summary, in a sample of 9 participants with bipolar disorder I, we were not able to find clear evidence of lunar cycling in sleep and mood patterns. Previous reports of lunar cycling in sleep in healthy participants focused on the top quartile of the sample (Casiraghi et al. 2021), indicating that the majority of individuals may not display systematic changes in sleep in association with the lunar cycle. To our understanding, all reports of lunar cycling in bipolar disorder, including our own, were limited to rapid cycling bipolar patients (Wehr 2018; Burgess et al. 2021; Avery et al. 2018). In our sample two participants were rapid cycling (P3, P5) which is consistent with the reported prevalence of rapid cycling (Kupka et al. 2003). P3 displayed consistently stable sleep and mood patterns, whereas P5 displayed later sleep onset only near the full moon but otherwise no consistent changes in total sleep time or mood. Our results are generated from a small sample but suggest the majority of people living with bipolar disorder do not display changes in sleep and mood in association with the lunar illuminance cycle. Such patterns are likely to be limited to a subset of individuals with a history of rapid cycling. Further study of lunar cycling should focus on those with rapid cycling bipolar disorder, and in a larger sample with a daily assessment of sleep and mood across a full year to better assess seasonal effects. Light sensitivity and any past response to light treatment in lunar cyclers should be also examined.

## Data Availability

The datasets generated and/or analyzed during the current study are not publicly available due to privacy restrictions, but are available from the Prechter study on reasonable request.
